# Supplementary material for: The interactome of intact mitochondria by cross-linking mass spectrometry provides evidence for coexisting respiratory supercomplexes
Source: Mol Cell Proteomics. 2017 Dec 8;17(2):216–32. doi: 10.1074/mcp.RA117.000470 (PMC5795388; doi:10.1074/mcp.RA117.000470)
Supplement: Supplemental Data [file supp_RA117.000470_133922_0_supp_23978_fzffwf.zip › spectra_annotation/mito_spectra_annotation/415-1-5-1-9-1.pdf]

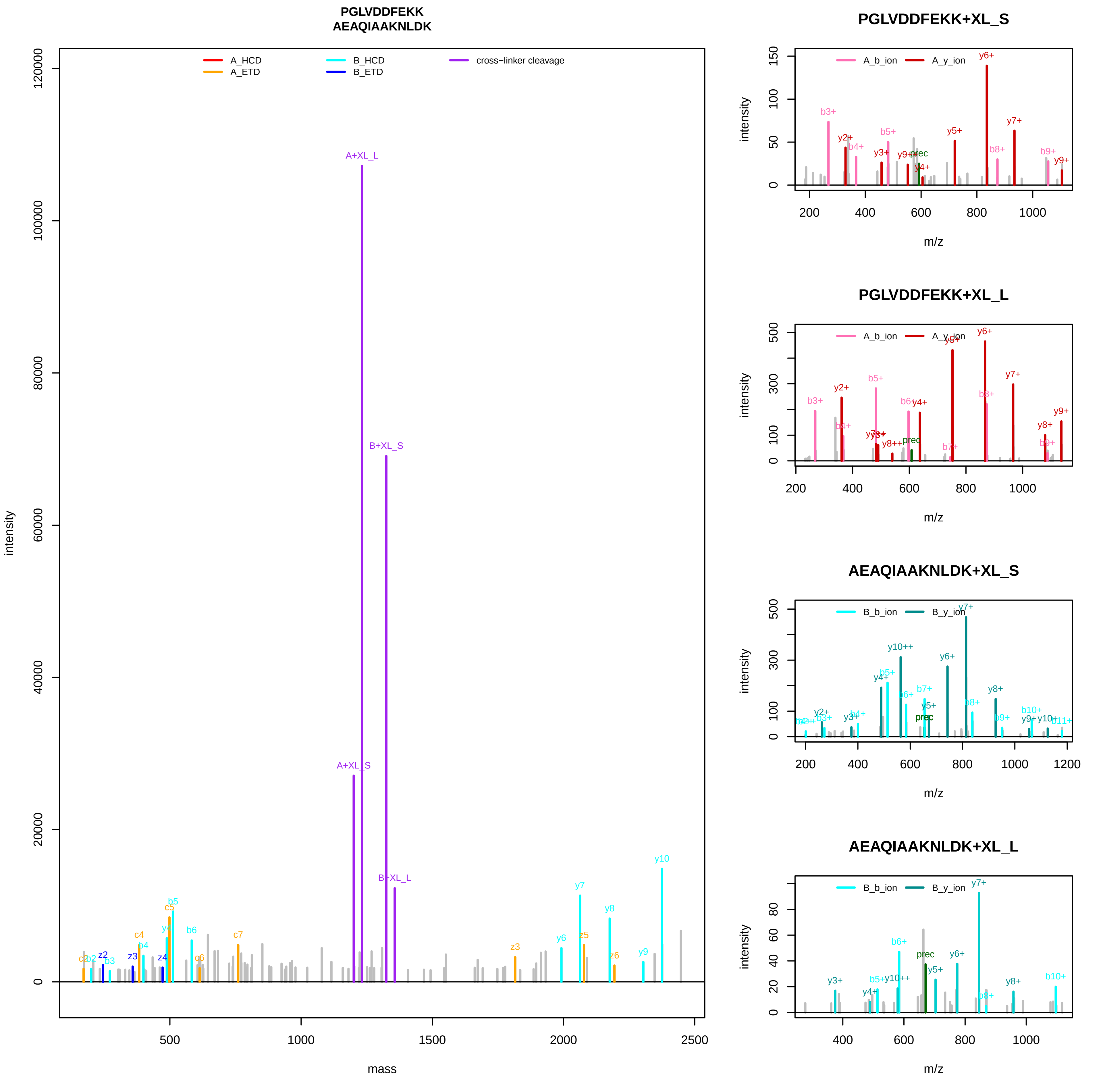

PGLVDDFEKK
AEAQIAAKNLDK
A_HCD
A_ETD
B_HCD
B_ETD
cross−linker cleavage
A+XL_L
B+XL_S
A+XL_S
B+XL_L
y10
y7
y8
y9
y6
z5
z6
z3
c7
c6
b6
b5
c5
y4
c4
b4
z4
z3
b3
z2
b2
intensity
mass
0
20000
40000
60000
80000
100000
120000
500
1000
1500
2000
2500
PGLVDDFEKK+XL_S
A_b_ion
A_y_ion
y6+
b3+
y7+
y2+
b5+
b4+
y5+
y3+
y9+
prec
b8+
y4+
b9+
y9+
intensity
m/z
0
50
100
150
200
400
600
800
1000
PGLVDDFEKK+XL_L
A_b_ion
A_y_ion
y6+
y5+
b5+
y2+
y7+
b3+
b6+
y4+
b8+
y9+
y8+
b4+
y3+
y8++
prec
b7+
b9+
intensity
m/z
0
100
300
500
200
400
600
800
1000
AEAQIAAKNLDK+XL_S
B_b_ion
B_y_ion
y7+
y10++
y6+
y4+
b5+
b7+
b6+
y8+
y5+
b8+
y2+
b4+
b3+
y3+
prec
b10+
b9+
y9+
y10+
b11+
intensity
m/z
0
100
300
500
200
400
600
800
1000
1200
AEAQIAAKNLDK+XL_L
B_b_ion
B_y_ion
y7+
b6+
prec
y6+
y5+
y3+
b5+
y10++
y4+
y8+
b10+
b8+
intensity
m/z
0
20
40
60
80
400
600
800
1000
